# Patient-Centered Leadership and Co-Design of Services for Breast Cancer Program in Nicaragua

**DOI:** 10.3390/ijerph22101482

**Published:** 2025-09-25

**Authors:** María Esther Suárez, Karen Vanessa Herrera, Alma Celeste Avilés, Gonzalo Granados, Ena Patricia García, Chepita Rivera

**Affiliations:** 1 Gynecology and Obstetrics Service, Military Hospital, Managua 12087, Nicaragua; maria.suarez@hospitalmilitar.com.ni; 2 Predicate AI Labs, Managua 14172, Nicaragua; 3 Maternal and Child Department, Military Hospital, Managua 12087, Nicaragua; jmaternoinfantil@hospitalmilitar.com.ni; 4 Mastologist, Military Hospital, Managua 12087, Nicaragua; gonzalo.granados@hospitalmilitar.com.ni; 5 Breast Unit, Military Hospital, Managua 12087, Nicaragua; voluntariado1.acpf@hospitalmilitar.com.ni (E.P.G.); voluntariado2@hospitalmilitar.com.ni (C.R.)

**Keywords:** breast cancer, patient leadership, co-design, person-centered care

## Abstract

**Introduction:** The direct participation and leadership of breast cancer patients in program design and implementation can facilitate a nuanced understanding of how individuals perceive and address challenges within their unique contexts. To achieve truly patient-centered care, patients must be formally integrated as a critical component of multidisciplinary healthcare teams. **Materials and Methods:** A descriptive, retrospective observational study was conducted. A group of 122 patients (“Breast Unit”) was formed and actively implemented initiatives related to education, prevention, peer support, co-design, and institutional guidance over two years. A second group, consisting of 466 individuals diagnosed with and treated for breast cancer, served as the Beneficiary Patient group. From the “Breast Unit”, 93 patients responded to a structured interview about their experience. **Results:** The Breast Unit group developed strong leadership skills and collaborated with the multidisciplinary healthcare team to improve care workflows, contributing at both strategic and operational levels. In total, 97% of patients received their first oncology evaluation within 48 h, ensuring timely intervention. The percentage of early-stage breast cancer diagnoses improved, from 67% to 76%. Furthermore, patients who participated in the support group reported no new diagnoses of clinical depression in the past two years. **Conclusions:** By elevating the patient voice into a substantive role within leadership, strategic planning, and co-design initiatives, healthcare systems can advance a more equitable, effective, and sustainable model of care. Integrating patient contributions with multidisciplinary collaboration is essential for optimizing care processes, improving clinical outcomes. It strengthens the person-centered culture, impacting on a personal and organizational level.

## 1. Introduction

A cancer diagnosis is a life-altering event with profound social, emotional, and physical and psychological implications that affect the quality of life [1,2]. Addressing these dimensions is essential for delivering holistic, patient-centered care that reflects a commitment to working for and with patients, making the system easy for them to access what they need [3,4].

Central to this approach is co-design, a collaborative process in which patients actively contribute to shaping their care experiences. At the Dr. Alejandro Dávila Bolaños Military Teaching Hospital (HMEADB), the establishment of the “Breast Unit” exemplifies this model. From its inception, the unit integrated patients as co-designers, ensuring their voices informed service delivery, educational materials, and support structures.

Co-design can be understood as encompassing the concepts of empowerment and social participation, consistent with Paulo Freire’s methodology for transforming reality [5]. International experience and multiple studies have demonstrated that participatory methods in healthcare improvement enable patients to actively engage in the co-production of services in cancer care, thereby fostering positive patient experiences and enhancing the quality of clinical practice among healthcare professionals [6,7].

Out of this co-design process emerged the role of the patient leader, a term used to describe individuals with lived experience of breast cancer who take on active roles in supporting, educating, and advocating for newly diagnosed patients and also contribute at strategic and leadership levels. Their involvement extended to planning social events, fostering community, and co-developing emotional and educational resources.

While the primary focus of this study is on the impact of patient leadership and peer support, it is important to acknowledge the broader multidisciplinary framework within which the Breast Unit operates. Multidisciplinary care—bringing together oncologists, surgeons, psychologists, and other specialists—has been shown to improve adherence to clinical guidelines, treatment outcomes, and the quality of decision-making [8,9]. Although this study does not directly measure the impact of multidisciplinary collaboration, it remains a foundational element of the unit’s comprehensive care model, particularly in cases where treatment decisions significantly affect quality of life [10].

This study examines the outcomes of an integrated, patient-led model, emphasizing the substantive role patients assumed in leadership, strategic planning, and co-design initiatives aimed at enhancing healthcare systems across multiple levels.

## 2. Materials and Methods

As a tertiary care hospital and national referral center, the Dr. Alejandro Dávila Bolaños Military Teaching Hospital (HMEADB) manages the largest number of oncology patients within the Social Security system. Notably, breast cancer represents the primary oncological diagnosis at this facility [11].

An observational descriptive study was conducted. The study population included two distinct groups: the first group was the Breast Unit recruited patients. This group consisted of 122 patients who, over two years, actively implemented initiatives and activities related to education, prevention, peer support, and institutional support.

The second group, designated as Beneficiary Patients, consisted of 466 individuals. These patients, diagnosed and treated for breast cancer at the Military Hospital, received the benefits of all strategies executed by the multidisciplinary team, yet they were not enrolled in the Breast Unit. While this paper centers on the first group, the inclusion of the Beneficiary Patients was solely for monitoring the impact of the initiatives.

Implementation of the Breast Unit was guided by the Patient-Centered Care framework [12] and experience-based co-design [13]. Within healthcare, co-design denotes a collaborative partnership between patients, caregivers, and staff aimed at service improvement. This approach underscores the pivotal role of patients in multidisciplinary management.

**Figure A1:**
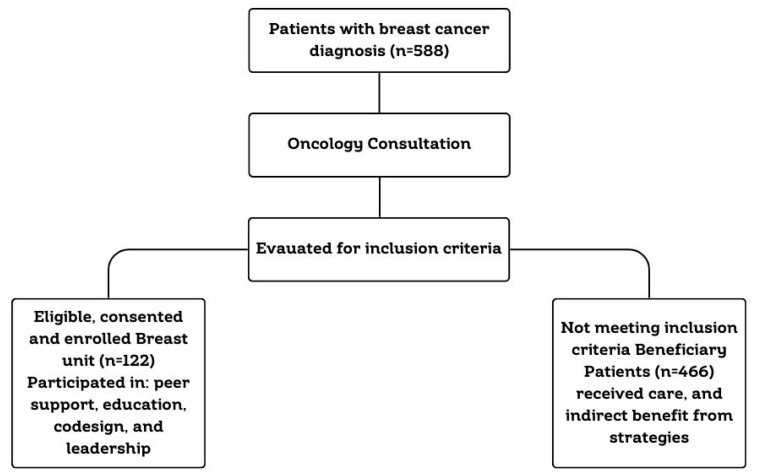
Recruitment process.

Patient recruitment for the Breast Unit was initiated in June 2022. Strategies were implemented for two years before the measurement of impact. The selection criteria for patient inclusion were as follows: Patients with a current diagnosis or survivors of breast cancer, Patients receiving exclusive treatment at the Military Hospital, Patients currently undergoing treatment or who have completed their treatment, Patients available for at least four hours weekly to support initiatives, Availability of a WhatsApp platform, Commitment to providing fluid and respectful support and communication, both in-person and virtually. (Figure A1) Patients signed an informed consent form and committed to follow the regulations for virtual and in-person preparticipation, which expert patients and medical specialists moderated.

The Breast Unit’s implementation strategy comprised both in-person and virtual components. In-person gatherings facilitated training, coordination, socialization, and collaborative efforts. Training specifically addressed nutrition, psychology, and emotional management, empowering patients not only to provide mutual support but also to cascade this knowledge.

For virtual engagement, WhatsApp and Zoom platforms were utilized due to their user-friendliness and patient accessibility, enabling coordinated meetings orchestrated by a patient leader. This fostered a crucial environment for patient empowerment, promoting reciprocal support, encouragement, enhanced hope, and a sense of companionship.

The Breast Unit integrated patients and a multidisciplinary team of qualified physicians, including Mastologists, Medical and Surgical Oncologists, Psychologists, Nutritionists, Physiotherapists, and Plastic Surgeons. These specialists underscored the group’s aims: to encourage disease acceptance, clarify patient doubts and fears, and participate in health promotion and educational activities.

The responsibilities of the participating members varied based on their time availability and the activities in which they felt most comfortable engaging. These included:

- Personal conversations between patients, calls to support each other.
- Attending weekly educational sessions to become permanent educators for their peers.
- Participating in technical committees to enhance patient safety and quality of care.
- Collaborating in the development and revision of educational materials, policies, and web resources.
- Providing recommendations to support the prevention of medical errors.
- Promoting activities related to Breast Cancer Awareness Month (October) within the institution, coordinating with and involving the Breast Unit’s support group.
- Organizing an activity commemorating Nicaraguan Mother’s Day (May 30th).

The participatory process included three key phases:

- Inspiration: Through structured meetings with hospital directors and multidisciplinary teams, patients shared their lived experiences, challenges, and unmet needs, providing critical insights into the realities of breast cancer care.
- Ideation: Patient feedback was instrumental in generating innovative ideas and practical solutions aimed at improving medical assistance, psychosocial support, and care coordination for breast cancer patients.
- Implementation: Patients assumed leadership roles in executing health strategies, ensuring that interventions were grounded in real-world relevance and tailored to the diverse needs of the breast cancer community.

Co-design activities aimed to provide a leading role that recognizes patients as protagonists within the framework of the person- and family-centered care policy [12,13] and included three levels: Firstly, patients collaborated in the co-design of services by contributing to the development of educational activities, psychosocial support programs, communication strategies, and community awareness events. Secondly, they actively participated in the co-design of service delivery, providing input on the organization of care pathways, reduction in waiting times, review of educational materials, and advising on improvements to hospital infrastructure, including the planning of a new oncology care facility. Thirdly, their contributions were integrated into the institutional framework of person- and family-centered care through formal participation in technical committees alongside hospital leadership and involvement in strategic planning meetings.

The second group included all breast cancer patients diagnosed and treated at the Military Hospital between 2022 and 2024 who were beneficiaries of all strategies, but were not enrolled in the Breast Unit. Follow-up data for these patients were obtained through medical record review.

### 2.1. Data Analysis

Data analysis for this initiative was structured into three key domains: communication efficiency, patient-reported experiences, and clinical outcomes.

Key indicators were established to monitor the impact of the strategy after the multidisciplinary health team was supported by volunteer patients enrolled in the “Breast Unit”. These indicators included the efficiency of virtual communication, as analyzed by response times to patient inquiries in the virtual chat using the Chat Analyzer application [14]. This application allowed us to quantify response times in the virtual chat, focusing on metrics such as average response time, frequency of interactions, and engagement between patient leaders and the healthcare team.

Communication on the WhatsApp platform was monitored and directed by patient leaders, who addressed inquiries from other breast cancer patients. These consultations did not pertain to medical or treatment aspects. Instead, they focused on upcoming activities, shared educational materials, recommendations for physical and emotional well-being, and guidance on how to support patients during challenging times. The healthcare team was present in the group to provide support to the patient leaders and to foster teamwork, reinforcing content that required further elaboration.

An online survey was administered to Breast Unit participants using Google Forms, selected for its accessibility and ease of use among our patient population. The instrument consisted of a total of 13 items, divided into two main sections: a socio-demographic and experiential questionnaire, and the second section evaluated depression symptoms. The first section aimed to gather sociodemographic data and assess participants’ experiences within a breast cancer support group. It included questions on time since breast cancer diagnosis, perceived level of support from the breast unit group, emotional and psychological impact of group participation, learned strategies to treatment challenges, degree of support among patients in the virtual group, usefulness of advice among patients regarding healthcare, and strengthening knowledge about the disease. These items were multiple-choice, allowing participants to select predefined options. However, each question included an open-ended response line, allowing participants to elaborate or share additional insights if desired.

The second section assessed depressive symptomatology through items intentionally designed to facilitate patient comprehension. Participants were asked to report whether, within the past two years, they had experienced sadness that interfered with their family, social, or professional life; persistent feelings of hopelessness; or recurrent thoughts of death. The section also assessed the perceived need for psychological support or pharmacological treatment to address mental health concerns. Responses were structured as binary (yes/no), with an additional open-ended field for comments.

The confidence level selected for the study was 95%, with a margin of error of 5%. A design effect (DEFF) of 1 was applied, assuming simple random sampling without clustering. Based on these parameters, the required sample size was determined to be 92 participants. This calculation was performed using the OpenEpi version 7.2.6 online tool for sample size estimation, which applies the following formula:

$$n=\frac{\mathrm{DEFF}\cdot N\cdot p\left(1-p\right)}{\frac{d^{2}}{Z_{1-\alpha /2}^{2}}\cdot \left(N-1\right)+p\left(1-p\right)}$$

where

- *n* = required sample size
- *N* = population size
- *p* = expected frequency (proportion)
- d = desired precision (margin of error)
- *Z* = Z-score corresponding to the selected confidence level
- DEFF = design effect

Prior to full deployment, the survey was piloted with 20 breast cancer patients to ensure clarity, relevance, and cultural appropriateness. The final version was distributed electronically via WhatsApp to all 122 participants in the Breast Unit, of whom 93 randomly agreed to respond to the survey, resulting in a response rate of 76 percent. This sample size falls within the required sample size for a 95% confidence level, according to the calculation performed using OpenEpi (*n* = 92). Therefore, the results retain statistical validity.

The remaining 29 patients did not complete the survey, primarily due to personal health-related challenges. Nevertheless, statistical validity was ensured based on the previously calculated sample size. Informal follow-up and available demographic data indicated that non-respondents did not differ significantly from respondents with respect to age, treatment status, or duration of participation in the Breast Unit.

For the second group, referred to as the beneficiary group, three evaluative indicators were established. The primary indicator was the proportion of early-stage breast cancer diagnoses, classified as an impact measure and aligned with Patient-Reported Outcome Measures (PROM) [15]. Early-stage breast cancer was defined as cases identified at stages 0, I, or IIA, in accordance with the staging criteria outlined by the American Joint Committee on Cancer [16,17]. Two additional indicators were employed to assess timeliness of care: (1) the interval between receipt of histopathological results and the initial consultation with an oncologist, and (2) the duration between the oncologist consultation and commencement of treatment. Data for these indicators were extracted through a systematic review of patient medical records. The collected data were subsequently organized in Microsoft Excel, and the corresponding percentage values for each indicator were calculated and reported.

### 2.2. Ethical Considerations

This study adhered to the Declaration of Helsinki [18], and it was approved by the Ethics Committee for Research at the Military Hospital (code: SCEHM2024-12). All patient data has been processed while respecting anonymity. Participating patients were informed about the use of their interactions in the support group for research purposes, and their informed consent was requested for data collection and analysis.

## 3. Results

Two years after its initiation, the Breast Unit formed a dedicated support group with 122 recruited patients. These patients significantly assisted healthcare staff by contributing to educational programs, strategic initiatives, social activities, and the design and implementation of strategies to improve patient care flow. Another group of 466 patients benefited from the contributions of these volunteers in their roles as counselors and educators, and they facilitated feedback mechanisms to ensure timely healthcare provision.

Most participants were between 30 and 50 years old (36.55%) or 50 and 70 years old (52.68%). The majority, 76%, were from the capital, while 24% resided in other departments of the country (Table 1).

### 3.1. Patient Leadership

The patients actively participated in designing and executing various activities, including educational sessions, relaxation workshops, and leadership meetings. Their defined roles and responsibilities included supporting leadership in technical meetings to develop and evaluate optimal breast cancer care plans, as well as collaborating with the hospital’s multidisciplinary team on annual event planning.

A significant outcome of this initiative was the establishment of their own board of directors. This board assumed organizational and managerial responsibilities within the group and actively collaborated with the hospital’s multidisciplinary team in planning activities and developing intervention strategies.

One of the foundational activities of the Breast Unit is to empower the newly recruited patients, providing them with an adequate level of knowledge on breast cancer care, treatment, and support. In this regard, through a patient-centered approach, the board assigned roles according to the level of experience or interest of participants:

1. Patient as a group leader: Patients with extensive diagnosis and treatment experience who mentored new members. This fostered immediate trust and relatability that professional healthcare providers, despite their expertise, cannot always replicate. This shared experience created a safe space for open dialogue.
2. Talk facilitators: Patients trained in specific topics who shared information with peers who promoted improved adherence to medications, earlier symptom recognition and management, lifestyle modifications, and follow-up appointments. They profoundly influenced the knowledge, confidence, and active participation of their peers.
3. Event coordinators: Responsible for organizing and managing special activities, such as Breast Cancer Awareness Month events. This was far more than an administrative delegation; it was a strategic investment in people-centered care. By leveraging their unique insights and passion, these patient leaders significantly enhanced awareness, built vital community support, and empowered their peers in their self-care.
4. Advocacy voices: Group representatives who participated in forums, conferences, and outreach activities both within and outside the hospital. These patients were the embodiment of people-centered care in action. They ensure that the healthcare system was not just treating a disease but truly serving the individuals living with it, leading to more relevant, accessible, and ultimately, more effective care and improved outcomes. The patient voice was present in leadership sessions for feedback.

### 3.2. Co-Design and Human-Centered Innovation

Patients collaborated with the hospital’s leadership to develop annual event plans, enhance quality improvement initiatives for breast cancer patient flow, and evaluate the strategic and operational annual plan. Their activities also extended to organizing relaxation workshops and religious gatherings. The program actively encouraged inclusive participation, acknowledging the diverse backgrounds and educational levels of the patients involved.

Patients from the breast unit were engaged and involved in the co-design of a new oncology building’s infrastructure, along with the associated patient care pathways for the new department at the Hospital.

Another important result highlights their role as primary educators. The group provided counseling and support, both through in-person sessions at the hospital and virtually. They also designed and executed educational talks in person, via social media groups, and the institution’s YouTube channel.

### 3.3. Multidisciplinary Collaboration Enhanced by Patient Insight

During numerous meetings with medical teams, patients consistently highlighted the need to optimize appointment waiting times. In response, the multidisciplinary team implemented immediate notifications for urgent cases and scheduled dedicated meetings to facilitate case discussions.

Additionally, based on insights from these joint sessions, an alert system was introduced in the hospital’s electronic records to flag BI-RADS 5 cases (indicating a 95% probability of being cancerous). This measure served as an extra reminder for all consultants, ensuring a more efficient and expedited care pathway for breast cancer patients.

Following the implementation of patient-centered interventions, 97% of patients received their initial oncology evaluation within 48 h of obtaining their histopathological diagnosis, thereby facilitating timely clinical decision-making. Furthermore, the revised care model significantly accelerated the initiation of treatment, whether medical, surgical, or a combination of both, with 97% of patients commencing therapy within seven days of their first oncology consultation. Prior to these improvements, patients typically faced waiting periods ranging from three weeks to one month, underscoring the substantial impact of the redesigned care pathway.

Another notable outcome following the implementation of educational strategies, supported by patients from the Breast Unit, was the improvement in early breast cancer detection rates. In 2022, the year the Breast Unit was established, the detection rate was 67%. This figure subsequently increased to 76% in both 2023 and 2024 (Table 2).

### 3.4. Psychosocial Impact and Emotional Resilience

A key foundation for success was maintaining a seamless flow of communication with the patients. To assess communication within the WhatsApp group, it was found that 83% of patient inquiries received a response within the first five minutes, while the remaining 17% were answered within five to ten minutes. The multidisciplinary team consistently moderated these interactions to ensure the accuracy and quality of the information provided (Table 3).

When asked about the personal impact of participating in and collaborating within the group, 87% of patients reported feeling highly supported, and 99% found peer-to-peer advice beneficial for their healthcare. Additionally, 91.4% stated that their knowledge had been strengthened through the group, while 80.6% expressed that it provided significant support in coping with difficult moments (Table 4).

One of the most impactful outcomes of this collaborative model was the creation of The Pink Book: Coloring with Green Hope, a collective testimony authored by 11 breast cancer patients. This resource was designed to support newly diagnosed individuals and their families, offering guidance across several domains:

- Understanding Your Diagnosis: Simplified explanations to help patients prepare for treatment, manage side effects, and recognize warning signs.
- Mental Health and Emotional Strength: Strategies to build resilience and maintain psychological well-being.
- Family and Social Support: Emphasis on the importance of a strong support network in navigating the cancer journey.
- Spirituality as a Refuge: Reflections on how faith and personal beliefs can provide comfort and peace during treatment.

Launched on World Breast Cancer Prevention Day, the book was disseminated widely through television, print media, and digital platforms, achieving national reach and raising public awareness about breast cancer. It promoted the importance of self-examination and timely medical attention, and was distributed free of charge to ensure accessibility.

## 4. Discussion

The operational model of the Breast Unit illustrates exemplary leadership practices within a healthcare context. To promote long-term sustainability and strategic coherence, a formal governing body was instituted, responsible for organizing the group and delineating roles and responsibilities. A collaborative environment emerged that not only enhanced operational efficiency but also strengthened interpersonal relationships within the unit (Table A1). Importantly, this model affirmed the role of patients as central agents in care delivery, consistent with the principles of person- and family-centered care [12].

The co-design process within the Breast Unit extended beyond conceptual discussions and was operationalized across three complementary levels: co-designing services, co-designing service delivery, and co-designing the framework for service provision. This multi-level approach positioned patients not merely as recipients of care, but as co-creators of care models, ensuring that both the content and the mode of delivery reflected their needs and perspectives. Previous publications suggest that co-design methodologies hold significant potential to make patient-centered services a reality [13].

Notably, breast cancer patients themselves assumed leadership roles in the design and implementation of the program. Their involvement was pivotal in fostering empathy and deepening the understanding of diverse health experiences. By integrating lived experiences into care planning, these patient leaders offered practical, experience-based solutions that transcended theoretical models [19]. This approach underscored the feasibility and value of patient collaboration, even in complex clinical contexts such as cancer care. Ultimately, the Breast Unit’s model exemplifies the principles of patient-centered care, anchored in human connection, empathy, and holistic support. It demonstrates that when patients are empowered as educators and leaders, they not only enhance the quality of care but also contribute to a more inclusive and responsive healthcare system.

Members of the Breast Unit evolved into empowered educators, actively shaping the landscape of breast cancer awareness and care. Their transformation was not only symbolic but instrumental in addressing a critical gap identified in prior studies [20] conducted in Nicaragua, which emphasized the urgent need for increased education among women regarding breast self-examination (BSE) as a tool for early detection of abnormalities due to the high prevalence even in young women [21]. This proactive educational approach aligns with global health recommendations that advocate for community-based interventions to improve breast health literacy, particularly in resource-limited settings.

Following diagnosis, the informational and emotional needs of patients and their families shift significantly. The Breast Unit responded by implementing structured educational initiatives that supported patients throughout their disease trajectory, enabling them to better understand their condition and explore effective strategies for disease management. This continuous education model fostered resilience and self-efficacy, empowering patients to actively participate in their care, consistent with previous literature [22].

Advisory patients played an active and strategic role in the design, planning, and execution of organizational initiatives, reinforcing the principle that patients are not merely recipients of care but integral contributors to healthcare teams. Their involvement in co-designing value-based strategies and disease-specific processes strengthened the concept of collaborative care and demonstrated the feasibility of patient-led innovation in clinical settings [23]. This empowerment fostered a strong sense of community and solidarity among individuals with shared experiences, particularly in the context of complex diseases such as cancer. The emotional and experiential bonds formed through this collaboration helped healthcare services identify and prioritize areas for improvement that were both meaningful and responsive to patient needs, as mentioned in previous experiences [24].

The approach adopted by the Breast Unit exemplifies the integration of Human-Centered Design (HCD) principles within healthcare systems, aligning with existing literature that identifies HCD as an effective framework for enhancing the quality and responsiveness of care delivery [25,26,27,28]. The initiative of the “pink book” beyond its educational value, highlighted the broader benefits of engaging patients who are currently undergoing treatment or have survived breast cancer. Their willingness to support others in similar circumstances enriched patient-provider communication and strengthened relational dynamics within care teams. Consistent with existing literature, this model of active patient involvement has been recognized as a cornerstone of equitable, effective, and sustainable healthcare systems [29,30].

Multidisciplinary collaboration is a cornerstone of effective breast cancer care, particularly in addressing delays in diagnosis and treatment that often result from fragmented approaches. A coordinated multidisciplinary team comprising Mastologists, oncologists, psychologists, nutritionists, physiotherapists, and plastic surgeons implemented a patient-centered protocol that prioritized BI-RADS 5 cases based on patients’ feedback. The high-suspicion diagnoses were flagged in the hospital’s electronic system, ensuring immediate evaluation within 24 h and facilitating streamlined care delivery [31].

The alert system significantly reduced diagnostic lag by synchronizing examination and consultation outcomes, allowing patients to bypass traditional bottlenecks. Studies have shown that such integrated workflows improve clinical efficiency, enhance adherence to treatment guidelines, and reduce recurrence rates [32,33]. Once a diagnosis was confirmed, patients were admitted to treatment within 48 h following administrative clearance, a practice aligned with international standards for timely cancer care.

Patient feedback played a pivotal role in refining this multidisciplinary model. Contributions from the Breast Unit extended beyond clinical input, involving patients in strategic planning and operational decisions. This participatory approach fostered psychosocial resilience and reduced stress, echoing findings from meta-analyses that highlight the benefits of peer-led support groups in managing chronic illnesses. These groups have been linked to improved self-efficacy, emotional well-being, and reduced healthcare utilization [34].

Despite these advancements, early detection remains a formidable challenge in resource-limited settings, with a high prevalence, where psychological, cultural, and infrastructural barriers often hinder timely diagnosis [35]. In this study, the increase in early-stage breast cancer detection from 67% to 76% underscores the impact of coordinated care and targeted education. Evidence from global initiatives supports the use of clinical breast exams and community health worker engagement as cost-effective strategies for improving outcomes in low-resource environments [36]. To further enhance care equity, future efforts should focus on adapting risk-based screening models to local contexts, investing in patient navigation systems, and expanding access to psychosocial support. These measures, when embedded within a multidisciplinary framework, can bridge systemic gaps and elevate the standard of breast cancer care across diverse populations.

Peer support in cancer care plays a pivotal role in enhancing patients’ quality of life by fostering mutual understanding, emotional resilience, and treatment adherence. Numerous studies have underscored the significance of such support systems, particularly in promoting participant empowerment and facilitating shared decision-making throughout the care continuum [37,38,39,40]. In this context, peer support—as demonstrated in the present study—serves not only as a source of emotional comfort but also as a mechanism for strengthening patients’ autonomy in decision-making and improving their self-regulation and coping strategies.

Patients actively engaged in breast unit programs consistently reported high levels of satisfaction with their care experiences. They described feeling heard, respected, and supported, with improved health literacy that contributed to a heightened sense of personal agency and psychological well-being during challenging phases of their treatment journey. This sense of empowerment was further reinforced by the opportunity to exchange experiences and insights with peers, which helped normalize their emotional responses and reduce feelings of isolation.

In the observed cohort, no patients reported symptoms of depression within the past two years, a finding that contrasts with existing literature [41,42]. Previous studies have established associations between strong social support systems and enhanced survival rates, increased adherence to treatment protocols, and reduced morbidity [43]. However, in the present study design, the evidence is insufficient to substantiate a definitive causal relationship.

Nevertheless, while these findings are promising, it is imperative to approach them with methodological rigor. To draw definitive conclusions regarding the mental health impact of peer support interventions, future studies must incorporate scales capable of establishing baseline psychological profiles and tracking changes over time. Instruments such as the Hospital Anxiety and Depression Scale (HADS), Patient Health Questionnaire (PHQ-9), and PROMIS Emotional Distress measures have been widely used in oncology settings and offer reliable metrics for assessing depression, anxiety, and emotional well-being [44]. Employing such tools will enable researchers to quantify outcomes more precisely and strengthen the evidence base for peer-led psychosocial interventions in cancer care.

## 5. Limitations

The present study is limited by the lack of a structured experimental or quasi-experimental framework to evaluate its effects on breast unit participants and the quality of care provided to future patients. To strengthen future evaluations, it is recommended that similar programs be evaluated through randomized controlled trials or matched comparison groups to more accurately assess outcomes, using a validated instrument. Additionally, the predominantly high educational background of the program’s participants may influence the generalizability of the findings. Finally, as this work was conducted in a single tertiary care center, contextual and institutional factors may influence applicability to other settings. Multicenter studies in diverse healthcare systems are recommended to expand the transferability of this patient-centered leadership model.

## 6. Conclusions

This study underscores the pivotal role of breast cancer patients as active contributors in the design and implementation of healthcare programs. Their leadership demonstrates that patient participation is not only feasible but also essential to achieve truly person-centered care. By integrating patients into decision-making processes and recognizing their lived experience as a source of expertise, healthcare systems can foster environments that strengthen emotional well-being, empowerment, and trust.

Furthermore, embedding patient leadership within strategic planning and multidisciplinary collaboration improves clinical outcomes while cultivating a culture of empathy, solidarity, and shared responsibility. These ripple effects extend beyond individual patient benefit, shaping institutional practices and reinforcing the value of patient voices as a cornerstone for building equitable, sustainable, and responsive models of cancer care.

## Figures and Tables

**Table 1:** Characteristics of patients in the breast unit support group.

Variable	Values	Number	Percentage (*n* = 93)
Age	Under 30 years old	8	11.6
	30 to 50 years old	34	36.6
	50 to 70 years old	49	52.7
	Over 70 years old	2	2.1
Origin	Managua	71	76.3
	Masaya	12	13
	Others	10	10.8
Educational level	University	82	88.2
	Secondary	11	12
Years since breast cancer diagnosis	Less than a year	19	20.4
	1 to 5 years	42	45.2
	5 to 10 years	21	22.6
	More than 10 years	11	12

Source: Survey of members of the Breast Unit group.

**Table 2:** Early Cancer Detection concerning Total Diagnosed Cases.

Years	Cases	Early Cancer Detection	Percentage
2022	92	62	67
2023	186	141	76
2024	188	143	76
Total	466	346	-

Source: Clinical records of patients diagnosed with breast cancer at HMADB.

**Table 3:** Response Time in Chat.

Time	Year 2022	Year 2023	Year 2024
Less than a min	16%	14%	16
From 1 to 5 min	46%	59%	67
From 5 to 15 min	38%	27%	17

Source: Chat analyzer based on a WhatsApp group.

**Table 4:** Personal impact of belonging to the support Breast Unit group.

Variable	Values	Numbers	Percentage (*n* = 93)
Degree of support among patients in the virtual group	None	2	2.1
	Little	3	3.2
	Moderate	7	7.5
	Very much	81	87.1
Usefulness of advice among patients regarding healthcare	Very useful	92	98.1
	Not useful	1	1.1
Strengthening knowledge about breast cancer	Yes	85	91.4
	No	8	8.6
Degree of support to face difficult moments	None	3	3.2
	Little	15	16.1
	Very much	75	80.6
Depression diagnosis in the last 2 years	Yes	0	0%
	No	93	100

Source: Survey of members of Breast Unit group.

## Data Availability

The original contributions presented in this study are included in the article. Further inquiries can be directed to the corresponding author.

## Appendix A

**Table A1:** Summary of the Breast Unit Program (2022–2024).

Components	Description
Institution	Dr. Alejandro Dávila Bolaños Military Teaching Hospital (HMEADB)
Primary Focus	Patient leadership, co-design, Breast cancer care and support
Study Groups	- Breast Unit Patients: 122 enrolled, active in initiatives - Beneficiary Patients: 466 treated at the hospital, not enrolled, but received benefits
Timeframe	June 2022–2024
Frameworks Used	- Patient-Centered Care, Patient leadership, co-design, breast cancer care and support
Inclusion Criteria (Breast Unit)	- Breast cancer diagnosis or survivor - Exclusive treatment at HMEADB - Available 4+ hrs/week - WhatsApp access and Commitment to respectful communication - Signed informed consent.
Implementation Strategy	- In-person: Training (nutrition, psychology, emotional management), coordination, socialization. - Virtual: WhatsApp & Zoom for meetings and support, - planning with patient leaders
Multidisciplinary Team	Mastologists, Oncologists, Psychologists, Nutritionists, Physiotherapists, Plastic Surgeons, and expert patients for feedback.
Patient Activities	- Peer support via calls and chats - Weekly educational sessions on virtual platforms and in person - Technical committee participation - Policy and educational material development - Breast cancer Awareness campaigns (October) - Mother’s Day celebration, - Advisors in leadership meetings
Co-design Contributions	- Meetings with hospital leadership, Survey design and implementation, and Educational planning. Feedback about infrastructure for the Oncology Department, Strategic and operational planning.
Impact Measurement (Breast Unit)	- Multidisciplinary collaboration (key feedback) - Virtual communication efficiency (Chat Analyzer) - 2024 survey on support, knowledge, coping, and depression diagnosis.
Impact Measurement (Beneficiary Group)	- PROM: % of early-stage breast cancer detection (Stages 0, I, IIA) - Time to medical attention post-diagnosis - Time to initial treatment post-consultation
